# Mycorrhizal association and its relation with pteridophytes

**DOI:** 10.3389/fmicb.2024.1406891

**Published:** 2024-07-11

**Authors:** Pratibha Kumari, Meenam Bhatia, Priti Giri, Prem Lal Uniyal

**Affiliations:** ^1^Department of Botany, Daulat Ram College, University of Delhi, Delhi, India; ^2^Department of Botany, Faculty of Sciences, University of Delhi, Delhi, India

**Keywords:** pteridophytes, mycorrhizal association, AM fungi, Glomeromycota, arbuscules, vesicles

## Abstract

Mycorrhizal association is one of the earliest and diversely distributed symbiotic associations on the Earth. This association helped early terrestrial plants to colonize the land by improved supply of nutrients like phosphate, nitrogen and zinc. It also helped plants to tolerate unfavorable soil conditions with increased water retention capacity, resistance to drought and pathogens. In return, fungi benefitted with carbon as their food source from the plants. More than 80% of terrestrial plants including pteridophytes, gymnosperms and angiosperms are reported to form arbuscular mycorrhizal (AM) association. Plants with root systems appeared on land during the Devonian period and many of them like pteridophytes still exist today. Various molecular and fossil studies confirm that the plants belonging to Ordovician-Devonian are associated with fungi, which are very similar to genus Glomus. AM association is very common in pteridophytes and the growth of its sporophyte and gametophyte is directly affected in the presence of AM association. Pteridophytes as early land plants with root systems have a very significant place in the plant kingdom. They have evolved and adapted to fill various habitats and facilitated early terrestrialization of other land plants by providing suitable niche with the help of AM fungi. In spite of pteridophytes being a very important plant group in the land system, very few reports are available on fungal-pteridophyte association. The present review is an effort to gather information about AM association in pteridophytes that might help in unraveling the evolution and significance of plant and fungi association.

## Introduction

Of all the essential relationships in nature, mycorrhiza is one of the most significant symbiotic relationships between plants and fungi. It is present in all soil types where plants can grow and facilitates the absorption of water and nutrients in the plants (Huey et al., 2020). About 80% of terrestrial plants distributed in different habitats show AM association (Quilambo, 2003). This mutual association played a very critical role in early terrestrialization and diversification of land plants. The alliance of AM fungi with plants is referred to as a bidirectional mutualistic symbiotic association, where both partners provide each other with nutrient resources (Kiers et al., 2011). Nearly 80% of nitrogen (N) and 100% of phosphorous (P) required for plant growth is provided by mycorrhizal fungi (Jakobsen et al., 1992; Leake et al., 2004; Smith and Smith, 2011; Hodge and Storer, 2015; Luginbuehl et al., 2017), whereas fungi utilize photosynthetically fixed organic carbon-based compounds from plants (Jakobsen and Rosendahl, 1990; Smith and Read, 2010). This association is not only restricted to nutrient exchange; it also helped plants to colonize land by increasing fitness and resistance and tolerance to drought, adverse soil conditions, and pathogenic microorganisms (Quilambo, 2003).

Mycorrhiza is a very primitive symbiotic association, and its origin can be traced back to the Ordovician period (475 Ma) when the first land plants colonized the Earth’s surface. These plants are thought to have an association with Glomeromycotina fungi that is very similar to modern AM association (Smith and Read, 2010). This association co-evolved with rootless plants to facilitate their transition from water to land and help in diversification (Pirozynski and Malloch, 1975; Redecker et al., 2000). Pteridophytes, among the first plants with roots that appeared on land during the Devonian period, are still flourishing. There is abundant evidence confirming that these pteridophytes form an association with Glomalean fungi and show presence of structures like vesicles (Read et al., 2000). Molecular studies also confirmed the emergence of Glomales during the same period as the evolution of land plants.

The knowledge about the diversity and function of fungal association in pteridophytes is inadequate (Strullu-Derrien and Strullu, 2007; Pressel et al., 2016; Lehnert et al., 2017). Mycorrhizal symbiosis occurs in both sporophytes (diploid) and gametophytic (haploid) phases of life. The vascular plants possess a root-bearing sporophytic phase, whereas gametophyte lacks roots and consists of rhizoids. In both phases, it is colonized by the same AM fungi (Winther and Friedman, 2008, 2009) and presence of AM fungi stimulates growth in both sporophyte and gametophyte (Turnau et al., 2005), but how this works in individual phases is still a question that needs to be answered.

## History of arbuscular mycorrhizal association

The mycorrhizal association is one of the most unique adaptations of land plants. This distinct association is found in 85% of living plant species (Brundrett, 2004; Smith and Read, 2008; van der Heijden et al., 2015; Brundrett and Tedersoo, 2018). Over time, distinct types of mycorrhizal association have evolved in different plants. AM association is one of the most widespread associations formed by fungi belonging to Glomeromycotina. These fungi are documented to colonize with roots and in some plants with rhizomes of vascular plants (Wang and Qiu, 2006; Smith and Read, 2008; Pressel et al., 2016). In nonvascular plants, like liverworts and hornworts, it is found to be present in the thallus (Strullu, 1985; Read et al., 2000; Selosse, 2005; Duckett et al., 2006; Pressel et al., 2010; Desireo et al., 2013). The AM association is widespread in land plants, but its geological record is sparse. The study of fossils plays an essential role in providing information on the evolutionary history of mycorrhizae and the extent of their association with plants, which helped them in early territorialization.

Fungal hyphae and spore of AM fungi were reported from 460 MY old sediments. However, these fossils are not directly found to be associated with plants (Redecker et al., 2000). The first-ever direct fossil evidence of plant-fungal association is documented from a 407 MY old Rhynie chert (Trewin and Rice, 2004), where multiple types of endomycorrhizal association are recorded in plants (Taylor and Krings, 2005; Strullu-Derrien et al., 2014; Taylor et al., 2015). The plants from Rhynie chert are small, and rhizoids are their rooting system (Kenrick and Strullu-Derrien, 2014). Aerial axes of *Aglaophyton majus* are reported to form arbuscules and are associated with fungi belonging to Glomeromycota (Remy et al., 1994). *Rhynia Gwynne-vaughan* shows the presence of vesicles (Boullard and Lemoigne, 1971; Karatygin et al., 2006), intercellular vesicles, and spores observed in *Nothia aphylla* (Krings et al., 2007). *Horneophyton lignieri* was found to be associated with fungi of Glomeromycotina (Strullu-Derrien et al., 2014). These early plant-fungal associations were named para mycorrhizas (Strullu-Derrien and Strullu, 2007) or mycorrhiza-like associations (Smith and Read, 2008) due to the lack of a proper root system. The roots evolved twice during the early evolution of vascular plants (Strullu-Derrien et al., 2014; Hetherington et al., 2016). However, knowledge about fungal symbiosis in early root systems is minimal. No record of fungal association in the roots of trees from the Middle Devonian period (398-359 MYA) is available (Morris et al., 2015). Harper et al. (2013) reported diversification in AM association in extinct pteridosperm *Glossopteris* from the Upper Permian of Antarctica (260–252 MYA). The associated fungi have septate hyphae considered Glomites (Taylor et al., 1995), different from aseptate fungi of Glomeromycotina. Associated fungi colonized with cortical cells in a helical pattern resembling *Paris*-type AM.

## Fungal structures formed by arbuscular mycorrhizal fungi in pteridophytes

Endomycorrhiza is one of the two types of mycorrhizal association found in plants. It is characterized by intra and intercellular hyphae in the root cortex, forming structures like arbuscules and vesicles. Arbuscules are highly branched, short-lived fungal hyphae essential for nutrient exchange, whereas vesicles are a storing structure. Vesicles exhibit lots of diversity in shape and size. Their size differs from 30 to 50 μ and shape varies from elliptical to spherical to irregular. Vesicles formed by *Glomus* sp. are generally spherical and elliptical, whereas rectangular and irregular-shaped vesicles are a feature of *Acaulospora* sp. (Ghosh et al., 2012). Among all endomycorrhizal fungal structures formed, hyphae appears to be first, followed by arbuscules and vesicles (Brundett, 2002).

Based on morphology, arbuscules are further divided into two types: *Arum* and *Paris*. *Arum*-type arbuscules are characterized by extensive dichotomized structures produced through the hyphae trunk in the cell lumen. In contrast, the *Paris*-type is characterized by hyphal coils in the root cortex (Dickson, 2004). Arbuscules are known to have ‘small tree’-like dichotomized morphology, whereas the *Paris*-type lacks such hyphal dichotomy. Harper et al. (2013) called *Paris*-type a mycorrhizal hyphae instead of arbuscules, although its function is the same as highly branched arbuscules. In addition to these two major morphological types, an *Intermediate*-type is also recognized, which shows the presence of fungal structures between the *Arum-and Paris-*type (Dickson, 2004) or the occurrence of either *Arum-and Paris-*type within the same species. A wide range of variation is observed in the degree of development from species to species (Smith and Smith, 1997). Many studies suggest that the type of arbuscular morphology depends on the host plants. Among all arbuscules morphology, *Paris-*type is widely distributed among land plants and reported in bryophytes, pteridophytes, gymnosperms, and flowering plant families (Simon et al., 1993; Taylor et al., 1995; Phipps and Taylor, 1996; Smith and Smith, 1997). In ferns, the *Paris*-type is considered the most dominant arbuscular type (Smith and Smith, 1997). These coiled intracellular hyphae and vesicles are also observed in *Aglaophyton*, a Devonian fossil (Kidston and Lang, 1921). Fernández et al. (2013) reported that *Intermediate-*type arbuscules tend more toward facultative species. *Paris*-type morphology is observed in genera *Pteris, Ophioglossum*, *Athyrium, Blechnum, Woodwardia, Alsophila, Cyathea, Equisetum, Osmunda, Campyloneurum, Dicranopteris, Lycopodium, Lepisorus, Drynaria, Microsorum Niphidium, Nephrolepis, Phlebodium, Pleopeltis, Polypodium,* and *Selaginella. Arum-*type is reported in a few genera, such as *Adiantum, Sticherus*, and *Microsorum*. *Intermediate*-type is seen in families *Allentodia, Adiantum, Blechnum, Cyathea, Diplazium, Grammitis, Hypolepsis, Microplepia, Pteridium, Arachniodes, Dryopteris, Polystichum, Dicranopteris, Lindsaea, Odontosoria, Sphenomeris, Stenoloma, Nephrolepsis, Huperzia, Angiopteris, Nephrolepis, Helminthostachys, Lycopodium, Cheilanthus, Doryopteris, Pellaea, Parahemionitis, Pityrogramma, Pteris, Campyloneurum, Drynaria, Microsorum, Pyrrosia, Selliguea, Salvinia, Lygodium, Selaginella,* and *Christella*. Many pteridophytes studied from different locations also show the presence of two types of arbuscule morphology (Table 1).

**Table 1:** Type of AM colonization in pteridophyte.

Family	Species	H	A	V	AM Type	References
Anemiaceae	*Anemia tomentosa*	+	+	+	I	Muthukumar and Prabha (2013)
	*A. phyllitidis*	+	+	+	NR	Parez et al. (2015)
Aspleniaceae	*Asplenium* sp.	+	−	+	NR	Heard (2006)
	*A. aethiopicum*	+	+	+	I	Muthukumar and Prabha (2013)
	*A. dalhousiae*	+	−	+	NR	Iqbal et al. (1981) and Nafees et al. (2019)
	*A. erectum*	+	+	−	I	Muthukumar and Prabha (2013)
	*A. gastonis*	+	−	+	NR	Krzyzanski et al. (2021)
	*A. incisum*	+	−	+	NR	Lee et al. (2001)
	*A. indicum*	+	+	+	I	Muthuraja et al. (2014)
	*A. lanceolatum*	+	+	+	I, P	Muthuraja et al. (2014)
	*A. tenuifolium*	+	+	+	I	Muthuraja et al. (2014)
	*A. varians*	+	+	+	I	Muthukumar and Prabha (2013)
Athyriaceae	*Allantodia chinensis* (Bak.) Ching	+	+	+	I	Zhang et al. (2004)
	*Athyrium asperum*	+	+	+	NR	Mishra et al. (1980)
	*A. bentamense*	+	−	+	NR	Mishra et al. (1980)
	*A. conili*	+	+	−	NR	Lee et al. (2001)
	*A. drepanopterum*	+	−	+	NR	Mishra et al., 1980
	*A. filix-foemina*	+	−	+	NR	Iqbal et al. (1981)
	*A. hohenakeranum* (Kunze) Moore	+	−	+	NR	Khade and Rodrigues (2002)
	*A. japonicum*	+	+	+	NR	Mishra et al. (1980) and Lee et al. (2001)
	*A. niponicum*	+	+	−	NR	Lee et al. (2001)
	*A. latifolium*	+	+	+	NR	Mishra et al. (1980)
	*A. wardii* (Hook.) Makino	+	+	+	P	Zhang et al. (2004)
	*Callipteris esculenta* (Retz.) J. Sm.	+	+	+	P	Zhang et al. (2004)
	*Diplazium donianum* (Mett.) Tard-Blot	+	−	+	P	Zhang et al. (2004)
	*D. lanceum* (Thunb.) Presl	+	+	+	P	Zhang et al. (2004)
	*D. polypodioides*	+	+	+	I	Muthuraja et al. (2014)
	*D. sylvaticum*	+	+	+	I	Muthuraja et al. (2014)
Blechnaceae	*Blechnum appendiculatum*	+	+	−	P	Parez et al. (2015)
	*B. occidentale*	+	+	+	I	Muthuraja et al. (2014)
	*B. orientale*	+	−	+	NR	Mishra et al. (1980) and Khade and Rodrigues (2002)
	*B. scheideanum*	+	+	+	P	Parez et al. (2015)
	*Brainea insignis*	+	+	+	NR	Mishra et al. (1980)
	*Woodwardia orientalis* Sw.	+	+	+	P	Zhang et al. (2004)
Cyatheaceae	*Alsophila firma*	+	+	+	P	Parez et al. (2015)
	*Cyathea bicrenta*	+	+	+	P	Parez et al. (2015)
	*C. divergens*	+	+	+	P	Parez et al. (2015)
	*C. gigantea*	+	+	+	I	Muthuraja et al. (2014)
Cystopteridaceae	*Cystopteris pellucida* (Franch.) Ching	+	+	+	P	Zhang et al. (2004)
Dennstaedtiaceae	*Hypolepsis glandulifera*	+	+	−	I	Muthukumar and Prabha (2013)
	*Dennstaedtia wilfordii*	+	+	−	NR	Lee et al. (2001)
	*D. hirsute*	+	+	−	NR	Lee et al. (2001)
	*Microplepia platyphylla*	+	+	+	I	Muthuraja et al. (2014)
	*Pteridium aquilinum*	+	+	+	I	Mishra et al. (1980), Muthukumar and Prabha (2013), and Muthuraja et al. (2014)
Dryopteridaceae	*Arachniodes amabilis*	+	+	+	I	Muthuraja et al. (2014)
	*A. rhomboidea* (Wall.) Ching	+	+	+	I	Zhang et al. (2004)
	*A. festina* (Hance) Ching	+	+	−	I	Zhang et al. (2004)
	*A. simplicior* (Makino) Ohwi	+	−	+	P	Zhang et al. (2004)
	*Crytomium falcatum* (L.f.) Presl	+	−	−	NR	Zhang et al. (2004)
	*Ctenitis* sp.	+	−	+	NR	Heard (2006)
	*Dryopteris amurensis*	+	+	−	NR	Lee et al. (2001)
	*D. fragrans*	+	+	−	NR	Lee et al. (2001)
	*D. fuscipes* C. Chr	+	+	+	I	Zhang et al. (2004)
	*D. blandfordii*	+	+	+	NR	Nafees et al. (2019)
	*D. lacera*	+	+	−	NR	Lee et al. (2001)
	*D. muenchii*	+	+	+	NR	Jaramillo et al. (2008)
	*Elaphoglossum petiolum*	+	−	−	P	Parez et al. (2015)
	*Paranema cyatheoides*	+	+	+	NR	Mishra et al. (1980)
	*Polystichum* sp.	+	+	+	I	Muthukumar and Prabha (2013)
	*P. craspedosorum*	+	+	−	NR	Lee et al. (2001)
	*P. lepidocaulon*	+	+	+	NR	Lee et al. (2001)
	*P. ordinatum*	+	−	−	P	Parez et al. (2015)
	*P. piceo-paleaceum*	+	+	+	I	Muthukumar and Prabha (2013)
	*P. tripteron*	+	+	−	NR	Lee et al. (2001)
	*P. tsusimense*	+	+	−	NR	Lee et al. (2001)
Equisetacae	*Equisetum arvense*	+	−	+	NR	Lee et al. (2001)
	*Equisetum hiemale* l.	+	+	+	P	Zhang et al. (2004) and Mane et al. (2019)
	*E. ramosissimum* Desf.	+	+	+	P	Zhang et al. (2004)
Gleicheniaceae	*Dicranopteris linearis*	+	+	+	I	Mishra et al. (1980) and Muthukumar and Prabha (2013)
	*D. dichotoma* (Thunb.) Bernh.	+	+	+	P	Lee et al. (2001) and Zhang et al. (2004)
	*D. linearis*	+	+	+	I	Muthuraja et al. (2014)
	*Gleichenia dichotoma*	+	−	+	NR	Khade and Rodrigues (2002)
	*G. japonica*	+	+	+	NR	Mishra et al. (1980)
	*Sticherus palmatus*	+	+	+	A	Parez et al. (2015)
Lindsaeaceae	*Lindsaea cultrata* (Willd.) Sw.	+	+	+	I	Zhang et al. (2004)
	*L. heterophyla*	+	+	+	NR	Khade and Rodrigues (2002)
	*Odontosoria chinensis*	+	+	+	I	Muthukumar and Prabha (2013)
	*Sphenomeris chinensis*	+	+	+	I	Mishra et al. (1980)
	*Sphenomeris chusana*	+	+	−	NR	Lee et al. (2001) and Muthuraja et al. (2014)
	*Stenoloma chusanum* (L.) Ching	+	+	+	I	Zhang et al. (2004)
Lycopodiaceae	*Huperzia hamiltonii*	+	+	+	I	Muthukumar and Prabha (2013)
	*Lycopodium clavatum*	+	+	+	I	Muthukumar and Prabha (2013)
	*L. cernuum*	+	+	+	P	Muthuraja et al. (2014)
	*Phlegmariurus mandiocanus*	+	−	+	NR	Krzyzanski et al. (2021)
Lygodiaceae	*Lygodium flexuosum*	+	+	+	NR	Mishra et al. (1980), Khade and Rodrigues (2002), and Bharti and Pravesh (2014)
	*L. japonicum*	+	+	+	I	Mishra et al. (1980) and Zhang et al. (2004)
	*L. microphyllum*	+	+	+	I	Muthuraja et al. (2014)
Marsileacea	*Marsilea* sp.	+	+	+	NR	Sarwade et al. (2012)
	*Marsilea quarifolia*	+	−	−	NR	Mane et al. (2019)
Marattiaceae	*Angiopteris evecta*	+	+	+	I	Muthukumar and Prabha (2013) and Muthuraja et al. (2014)
	*Marattia* sp.	+	+	−	P	Parez et al. (2015)
	*Angiopteris*	+	−	+	NR	Santhoshkumar and Nagarajan (2014)
Nephrolepidaceae	*Nephrolepis* sp.	+	+	+	NR	Sarwade et al. (2012)
	*N. auriculata*	+	+	+	I	Muthuraja et al. (2014)
	*N. multiflora*	+	−	+	I, P	Muthuraja et al. (2014)
	*Nephrolepis* sp.	+	+	+	I	Muthukumar and Prabha (2013)
	*N. cordifolia*	+	+	+	I	Muthukumar and Prabha (2013)
	*N. exaltata*	+	+	+	I	Muthukumar and Prabha (2013) and Mane et al. (2019)
	*N. falcata*	+	+	+	I	Muthukumar and Prabha (2013)
Oleandraceae	*Oleandra wallichii*	+	+	+	NR	Mishra et al. (1980)
Onocleaceae	*Matteuccia struthiopteris*	+	+	−	NR	Lee et al. (2001)
	*Onoclea sensibilis* var. *interrupta*	+	+	−	NR	Lee et al. (2001)
Ophioglossaceae	*Ophioglossum reticulatum*	−	+	+	P	Muthukumar and Prabha (2013) and Ghanta et al. (2016)
	*O. vulgatum* L.	+	+	+	P	Zhang et al. (2004) and Mane et al. (2019)
	*Botrychium viriginiarum*	+	−	+	NR	Mishra et al. (1980)
	*Botrychium daucifolium* Wall. Hook and Grev.	+	+	−	NR	Santhoshkumar and Nagarajan (2014)
	*B. lanuginosum* Wall.	+	−	+	P	Zhang et al. (2004)
	*B. ternatum* (Thunb.) Sw.	+	−	+	P	Zhang et al. (2004)
	*Helminthostachys zeylanica*	NR	+	+	I	Ghanta et al. (2016)
	*Sceptridium ternatum*	+	+	+	NR	Lee et al. (2001)
	*S. multifidum* var. *robustum*	+	+	−	NR	Lee et al. (2001)
Osmundaceae	*Osmunda claytonianum*	+	+	−	NR	Lee et al. (2001)
	*O. cloyioniana*	+	+	+	NR	Mishra et al. (1980)
	*O. japonica* Thumb.	+	+	+	P	Lee et al. (2001) and Zhang et al. (2004)
Plagiogyriaceae	*Plagiogyria adnata*	+	+	+	NR	Mishra et al. (1980)
Pteridaceae	*Aleuritopteris argentea*	+	+	+	NR	Lee et al. (2001)
	*Actiniopteris radiata*	+	+	+	NR	Muthukumar and Prabha (2013) and Mane et al. (2019)
	*Adiantum* sp.	+	+	+	A	Ghosh et al. (2012) and Ghanta et al. (2016)
	*A. andicola*	+	−	+	A	Parez et al. (2015)
	*A. capillus-veneri*	+	+	+	I, A	Iqbal et al. (1981), Muthuraja et al. (2014), Santhoshkumar and Nagarajan (2014), Mane et al. (2019), and Nafees et al. (2019),
	*A. caudatum* L.	+	+	+	NR	Santhoshkumar and Nagarajan (2014), Mane et al. (2019), and Nafees et al. (2019)
	*A. concicnnum*	+	−	+	P	Parez et al. (2015)
	*A. hispidulum*	+	+	+	I	Muthukumar and Prabha (2013) and Muthuraja et al. (2014)
	*A. incisum*	+	+	−	I	Muthukumar and Prabha (2013) and Muthuraja et al. (2014)
	*A. lunulatum* Burm.	+	−	+	I	Khade and Rodrigues (2002), Muthukumar and Prabha (2013), and Mane et al. (2019)

	*A. pedatum*	+	+	−	NR	Lee et al. (2001)
	*A. philippense*	NR	+	+	A	Ghanta et al. (2016)
	*A. radianum* (rq) Flickr	+	+	+	I, P	Muthukumar and Prabha (2013), Muthuraja et al. (2014), and Santhoshkumar and Nagarajan (2014)
	*A. trapeziforme*	+	−	+	P	Parez et al. (2015)
	*A. zollingeri* Mett ex Kuhn	+	+	−	I	Muthukumar and Prabha (2013) and Santhoshkumar and Nagarajan (2014)
	*Cheilanthus* sp.	+	+	+	NR	Ghosh et al. (2012)
	*C. bullosa* kunze	+	+	+	I	Muthukumar and Prabha (2013) and Santhoshkumar and Nagarajan (2014)
	*C. farinose*	+	+	+	I, P	Mishra et al. (1980), Muthuraja et al. (2014), and Mane et al. (2019)
	*C. mysorensis*	+	+	+	NR	Santhoshkumar and Nagarajan (2014)
	*C. opposite*	+	+	+	I, P	Muthuraja et al. (2014)
	*C. swartzii*	+	+	+	I	Muthukumar and Prabha (2013)
	*C. tenuifolia*	+	+	+	I	Muthuraja et al. (2014)
	*Coniogramme japonica*	+	−	+	NR	Lee et al. (2001)
	*Doryopteris concolor*	+	+	+	I	Muthuraja et al. (2014)
	*Hemionitis arifolia*	+	+	+	I	Muthuraja et al. (2014)
	*Onychium* sp.	+	−	+	NR	Ghosh et al. (2012)
	*Hemionitis arifolia*	+	−	+	NR	Santhoshkumar and Nagarajan (2014)
	*O. japonicum*	+	−	+	NR	Iqbal et al. (1981)
	*Pellaea concolor*	+	+	+	I	Muthukumar and Prabha (2013)
	*Parahemionitis cordata*	+	+	+	I	Muthukumar and Prabha (2013)
	*Pteridium aquilinum* (L.) Kuhn	+	+	+	P	Lee et al. (2001), Zhang et al. (2004), and Nafees et al. (2019)
	*Pteris* sp.	+	+	+	I	Ghosh et al. (2012) and Muthukumar and Prabha (2013)
	*P. aspericaulis* Wall.	+	+	+	P	Zhang et al. (2004)
	*P. argyraea*	+	+	−	I	Muthukumar and Prabha (2013)
	*P. biaurita*	+	+	+	I	Mishra et al. (1980), Muthuraja et al. (2014), and Santhoshkumar and Nagarajan (2014)
	*P. confuse*	+	+	+	I	Muthukumar and Prabha (2013)
	*P. catoptera*	+	+	+	I	Muthukumar and Prabha (2013)
	*P. cretica*	+	+	+	I	Lee et al. (2001), Muthukumar and Prabha (2013), and Nafees et al. (2019)
	*P. ensifolia*	+	+	+	NR	Mishra et al. (1980)
	*P. gongalensis*	+	+	+	I	Muthukumar and Prabha (2013)
	*P. multifida*	+	+	+	P	Lee et al. (2001) and Ghanta et al. (2016)
	*P. quadrianrita*	+	+	+	NR	Mishra et al. (1980)
	*P. pellucida*	+	+	+	I	Muthukumar and Prabha (2013) and Muthuraja et al. (2014)
	*P. vittata* L.	+	+	+	P	Mishra et al. (1980), Khade and Rodrigues (2002), Zhang et al. (2004), Martinez et al. (2012), Muthukumar and Prabha (2013), Ghanta et al. (2016), and Mane et al. (2019)
*P. wallichiana*	+	+	+	NR	Mishra et al. (1980)

	*Pityrogramma calomelanos*	+	+	+	I	Khade and Rodrigues (2002), Muthukumar and Prabha (2013), and Muthuraja et al. (2014)
*Scoliosorous* sp.	+	−	+	NR	Heard (2006)
Polypodiaceae	*Campyloneurum aglaolepis*	+	−	+	NR	Krzyzanski et al. (2021)
	*C. costatum*	+	+	−	P	Parez et al. (2015)
	*C. nitidum*	+	−	+	NR	Krzyzanski et al. (2021)
	*Crypsinus hastatus*	+	+	+	NR	Mishra et al. (1980) and Lee et al. (2001)
	*Drynaria quercifolia*	+	+	+	I, P	Muthukumar and Prabha (2013) and Muthuraja et al. (2014)
	*Grammitis attenuata*	+	+	+	I	Muthukumar and Prabha (2013)
	*Humata repens*	+	−	+	NR	Santhoshkumar and Nagarajan (2014)
	*Lemmaphyllum microphyllum*	+	+	+	NR	Lee et al. (2001)
	*Lepisorus nudus*	+	+	+	P	Muthuraja et al. (2014)
					NR	Muthuraja et al. (2014)
	*Microsorum hymenoides*	+	+	+	I	Muthukumar and Prabha (2013)
	*M. membranaceum*	+	+	+	I	Muthukumar and Prabha (2013)
	*M. punctatum*	NR	+	+	A	Ghanta et al. (2016)
	*Niphidium crassifolium*	+	+	+	P	Parez et al. (2015) and Krzyzanski et al. (2021)
	*Pecluma pectinatiformis*	+	−	+	NR	Krzyzanski et al. (2021)
	*Phlebodium aureum*	+	−	−	P	Parez et al. (2015)
	*P. pseudoaureum*	+	−	−	P	Parez et al. (2015)
	*Pleopeltis* sp.	+	−	+	NR	Heard (2006)
	*P. crassinervata*	+	−	+	P	Parez et al. (2015)
	*P. hirsutissima*	+	−	+	NR	Krzyzanski et al. (2021)
	*P. pleopeltifolia*	+	−	+	NR	Krzyzanski et al. (2021)
	*Polypodium* sp.	+	−	+	P	Parez et al. (2015)
	*P. lachnopus*	+	+	+	NR	Mishra et al. (1980)
	*P. lepidotrichum*	+	−	+	P	Parez et al. (2015)
	*Pyrrosia porosa*	+	+	+	I	Muthukumar and Prabha (2013)
*Selliguea montana*	+	+	−	I	Muthukumar and Prabha (2013)
Salviniaceae	*Salvinia molesta*	+	+	+	I	Muthukumar and Prabha (2013)
Selaginellaceae	*Selaginella* sp.	+	+	+	I, P	Khade and Rodrigues (2002), Muthukumar and Prabha (2013), and Muthuraja et al. (2014)
	*S. wightii*	+	+	+	I	Muthuraja et al. (2014)
	*S. bryopteris*	−	+	−	P	Muthukumar and Prabha (2013)
	*S. davidii* Franch.	+	−	+	P	Zhang et al. (2004)
	*S. doederleinii*	+	+	+	I	Muthukumar and Prabha (2013)
	*S. finitima*	+	−	+	I	Parez et al. (2015)
	*S. martensii*	+	−	−	I	Parez et al. (2015)
	*S. microphylla*	+	−	+		Krzyzanski et al. (2021)
	*S. moellendrofii* Hieron.	+	−	+	I	Zhang et al. (2004)
	*S. wightii*	+	+	+	I	Muthuraja et al. (2014)
Tectariaceae	*Tectaria coadunata*	+	+	+	I	Muthuraja et al. (2014)
Thelepteridaceae	*Ampelopteris prolifera*	+	+	+	NR	Bharti and Pravesh (2014)
	*Christella dentata*	+	+	+	I, P	Muthukumar and Prabha (2013), Muthuraja et al. (2014), and Khade and Rodrigues (2002)
	*C. parasitica*	+	+	+	I	Muthukumar and Prabha (2013) and Muthuraja et al. (2014)
	*Dictyocline wilfordii* (Hook.) J. Sm	+	+	+	I	Zhang et al. (2004)
	*Macrothelypteris torresiana*	+	+	+	I	Muthuraja et al. (2014)
	*Meniscium* sp.	+	+	+	I	Muthukumar and Prabha (2013)
	*Pseudocyclosorus xylodes*	+	+	+	I	Muthuraja et al. (2014)
	*Sphaerostephanos arbuscula*	+	+	+	I	Muthuraja et al. (2014)
	Thelypteris sp.	+	−	+	NR	Heard (2006)
	*T. aurita*	+	+	+	NR	Mishra et al. (1980)
	*T. ciliata*	+	+	+	NR	Mishra et al. (1980) and Muthukumar and Prabha (2013)
	*T. gracilascens*	+	+	+	NR	Mishra et al. (1980)
	*T. multibineota*	+	+	+	NR	Mishra et al. (1980)
	*T. novebonacensis*	+	+	+	NR	Mishra et al. (1980)
Cystopteridaceae	Cystopteris sp.	+	−	+	NR	Heard (2006)
Woodsiaceae	*Woodsia ilvensis*	+	+	+	NR	Lee et al. (2001)

H, Hyphae; V, Vesicles; A, Arbuscules; AM Type, Arbuscular Mycorrhizal Type; I, Intermediate type; P, Paris type; A, Arum type; NR, Not reported.

## Arbuscular mycorrhizal fungi in pteridophytes

Soil fungi are very diverse and are known to play an important role as belowground biota. They are known for their significant role in forest dynamics (Copely, 2000). These soil fungi are found to have associations with plants; among those association, AM association is one of the most primitive and crucial symbiotic associations. These fungi are known to improve soil nutrition and fertility by helping plants to uptake complex nutrients such as phosphorus, nitrogen, and zinc. Their association is well known for influencing plant diversity and community structure (van der Heijden et al., 1998; Read, 1999; O’Connor et al., 2002).

Around 317 species of AM fungi are reported based on morphological characters (International Culture Collection of Glomeromycota [CICG], 2020). These AM-forming fungi are classified under four orders: Archaesporales, Diversisporales, Glomales, and Paraglomales (Wijayawardene et al., 2020). They are known for their coenocytic mycelium, production of arbuscular, asexual modes of reproduction through sporogenesis at the hyphal tip, and formation of vesicles, which is a lipid-storing structure (Redecker and Raab, 2006; Schüßler and Walker, 2011). Arbuscules are standard structures in all AM-forming fungi, whereas the formation of vesicles is restricted to only some genera. Fungal genera such as *Gigaspora* and *Scutellospora* lack the ability to form vesicles (Redecker and Raab, 2006). Among all AM-forming fungi, *Glomus* is the most significant and commonly found AM fungi to be associated with every plant group. Numerous fossils and genetic studies confirmed Glomales association with vascular plants of the Devonian period. Glomales are associated with tropical pteridophytes (Read et al., 2000). Many workers also confirm the predominance of *Glomus* genera associated with pteridophytes. Apart from *Glomus*, other species belonging to *Acaulospora, Claroideoglomus, Entrophospora, Funneliformis, Gigaspora, Sclerocystis* and *Scutellospora* are also widely reported to form AM association with pteridophytes (Table 2).

**Table 2:** Diversity of AM fungi associated with pteridophytes.

Species	Glo	Giga	Acaul	Scl	Scut	References
*Adiantum lunulatum*	+	−	+	+	+	Khade and Rodrigues (2002)
*Athyrium hohenackeranum* Moore	+	−	−	−	+	Khade and Rodrigues (2002)
*Adiantum* sp.	+	+	+	−	−	Ghosh et al. (2012)
*Adiantum capillus*	+	−	−	−	−	Nafees et al. (2019)
*A. caudatum*	+	−	−	−	−	Nafees et al. (2019)
*Asplenium dalhousia*	+	−	−	+	−	Nafees et al. (2019)
*A. gastonis*	+	−	−	−	−	Krzyzanski et al. (2021)
*Athyrium aspera*	−	+	−	+	NR	Mishra et al. (1980)
*A. bentamense*	−	−	+	−	NR	Mishra et al. (1980)
*A. drepanopterum*	+	−	+	−	NR	Mishra et al. (1980)
*A. japonicum*	+	+	−	+	NR	Mishra et al. (1980)
*A. latifolium*	+	+	−	+	NR	Mishra et al. (1980)
*Blechnum orientale,*	+	−	−	−	−	Khade and Rodrigues (2002)
*B. orientale*	−	−	−	+	NR	Mishra et al. (1980)
*Bruinea insignis*	−	+	−	+	NR	Mishra et al. (1980)
*Botrychium viriginiarum*	+	+	−	+	NR	Mishra et al. (1980)
*Campyloneurum nitidum*	+	−	+	−	−	Krzyzanski et al. (2021)
*Cheilanthes* sp.	+	−	+			Ghosh et al. (2012)
*C. farinose*	+	−	−	+	NR	Mishra et al. (1980)
*Cycloporus exiensis*	+	−	+	+	NR	Mishra et al. (1980)
*Crypsinus hastatus*	+	−	−	+	NR	Mishra et al. (1980)
*Dryopteris blandfordii*	+	−	−	−	−	Nafees et al. (2019)
*D. elongate*	+	+	+	+	NR	Mishra et al. (1980)
*D. hirtipes*	−	−	+	+	NR	Mishra et al. (1980)
*D. nigra*	+	+	−	+	NR	Mishra et al. (1980)
*D. spersa*	+	+	+	+	NR	Mishra et al. (1980)
*Dricanopteris lincaris*	+	−	−	+	NR	Mishra et al. (1980)
*Gleichenia dichotoma*	+	−	+	+	+	Khade and Rodrigues (2002)
*Gleichenia japonica*	−	+	−+		NR	Mishra et al. (1980)
*Lindsaea heterophylla*	+	−	−	−	−	Khade and Rodrigues (2002)
*Lindsaya cuttrota*	+	+	−	+	NR	Mishra et al. (1980)
*Lygodium flexuosum*	−	−	−	+	−	Khade and Rodrigues (2002)
*L. flexnosum*	−	+	−	+	NR	Mishra et al. (1980)
*L. japonicum*	+	−	−	−	NR	Mishra et al. (1980)
*Microgramma vacciniifolia*	+	−	−	−	−	Krzyzanski et al. (2021)
*Niphidium crassifolium*	+	−	+	−	−	Krzyzanski et al. (2021)
*Oleandra wallichi*	−	−	−	+	NR	Mishra et al. (1980)
*Osmunda cloyioniana*	+	−	−	+	NR	Mishra et al. (1980)
Onychium sp.	+	+	−	−	−	Ghosh et al. (2012)
*Pityrogramma calomelanos*	+	+	−	−	+	Khade and Rodrigues (2002)
*Pteris* sp.	+	+	+	−	−	Ghosh et al. (2012)
*Pteris vittate*	+	−	−	+	−	Khade and Rodrigues (2002)
*Pteridium aquilinum*	+	+	−	−	−	Nafees et al. (2019)
*Pteris cretica*	+	+	−	−	−	Nafees et al. (2019)
*Pecluma pectinatiformis*	−	−	+	−	−	Krzyzanski et al. (2021)
*Phlegmariurus mandiocanus*	−	−	+	−	−	Krzyzanski et al. (2021)
*Pleopeltis pleopeltifolia*	−	−	+	−	−	Krzyzanski et al. (2021)
*P. hirsutissima*	−	+	+	−	−	Krzyzanski et al. (2021)
*Paranema cyatheoides*	+	_	+	+	NR	Mishra et al. (1980)
*Polystychium acubatum*	+	_	_	+	NR	Mishra et al. (1980)
*P. abericulatum*	−	−	−	+	NR	Mishra et al. (1980)
*P. semifertile*	+	−	+	+	NR	Mishra et al. (1980)
*Plagiogyra adnota*	−	+	−	+	NR	Mishra et al. (1980)
*Polypodium lachnopus*	−	−	+	+	NR	Mishra et al. (1980)
*Pteridium aquilivium*	−	−	−	+	NR	Mishra et al. (1980)
*Pteris bianrita*	+	−	−	+	NR	Mishra et al. (1980)
*P. quadrianrita*	+	−	−	−	NR	Mishra et al. (1980)
*P. vittate*	+	−	−	+	NR	Mishra et al. (1980)
*P. wallichiana*	−	−	+	+	NR	Mishra et al. (1980)
*P. ensifolia*	+	+	−	+	NR	Mishra et al. (1980)
*Syngramme vestita*	+	−	−	−	−	Ghosh et al. (2012)
*Sphenomeris chinensis*	+	−	−	+	NR	Mishra et al. (1980)
*Sellaginella* sp.	+	+	−	−	−	Khade and Rodrigues (2002)
*Thelypteris aurita*	+	−	−	+	NR	Mishra et al. (1980)
*T. ciliata*	+	−	−	+	NR	Mishra et al. (1980)
*T. gracilascens*	+	−	−	+	NR	Mishra et al. (1980)
*T. multibineota*	+	−	−	+	NR	Mishra et al. (1980)
*T. novebonacensis*	+	−	+	+	NR	Mishra et al. (1980)

Glo, Glomus; Giga, Gigaspora; Acaul, Acaulospora; Scl, Sclerocystis; Scut, Scutellospora; NR, Not reported.

Many AM fungal spore populations have been isolated from rhizosphere soil of pteridophytes, including *Acaulospora cavernata, A. colossica, A. foveata, A. gdanskensis, A. gedanensis, A. lacunosa, A. laevis, A. morrowiae, A. rehemii, A. scorbiculata,* and *A. thomii* (Khade and Rodrigues, 2002; Zhang et al., 2004; Muthuraja et al., 2014; Santhoshkumar and Nagarajan, 2014; Krzyzanski et al., 2021). *Claroideoglomus candidum, C. etunicatum,* and *C. luteum* (Muthukumar and Prabha, 2013; Krzyzanski et al., 2021), *Entrophospora infrequens* (Santhoshkumar and Nagarajan, 2014). *Funneliformis constrictum* and *F. geosporum* (Muthukumar and Prabha, 2013; Krzyzanski et al., 2021), *Glomus aggregatum, G. ambisporum, G. australe, G. botryoides, G. caledonium, G. convolutum, G. claroideum, G. desecticola, G. dimorphicum, G. etunicatum, G. fasiculatum, G. fistulosum, G. formosanum, G. globiferum, G. heterosporum, G. halon, G. hoi, G. intraradices, G. invermaium, G. macrocarpum, G. mosseae, G. monosporum, G. moaaeae, G. morphicum, G. microcarpum, G. multisubtensum, G. multicaulis, G. pustulatum, G. rubiformis,* and *G. versiforme* (Koske et al., 1985; Khade and Rodrigues, 2002; Zhang et al., 2004; Jaramillo et al., 2008; Ghosh et al., 2012; Martinez et al., 2012; Muthukumar and Prabha, 2013; Muthuraja et al., 2014; Ghanta et al., 2016; Nafees et al., 2019; Krzyzanski et al., 2021), *Gigaspora albida, G. candida, G. decipiens, G. margarita,* and *G. gigantea* (Khade and Rodrigues, 2002; Muthuraja et al., 2014; Santhoshkumar and Nagarajan, 2014; Nafees et al., 2019), *Sclerocystis pachycaulis, S. rubiformis,* and *S. taiwanensis* (Khade and Rodrigues, 2002; Muthuraja et al., 2014; Santhoshkumar and Nagarajan, 2014; Nafees et al., 2019), *Scutellospora heterogama, S. scutata, S. gregaria,* and *S. reticulata* (Khade and Rodrigues, 2002; Santhoshkumar and Nagarajan, 2014).

## Factors affecting arbuscular mycorrhizal association

Arbuscular mycorrhizal fungi show worldwide distribution and are found in almost all soil types (Mosse et al., 1981; Daniels Hetrick, 1984). Their distribution and function are extensively affected by various edaphic conditions such as soil composition, temperature, moisture, cation exchange capacity, pH, and anthropogenic abiotic and biotic stresses, including soil compaction and the presence of metals and pesticides. Adequate soil moisture and temperature play a positive role in the development of AM association.

Soil temperature is one of the key abiotic factors that plays an important role in AM fungal growth. Heinemeyer and Fitter (2004) reported the direct effect of soil temperature on the growth of mycorrhizal mycelium. With a rise in temperature from 12 to 20°C, there is a significant increase observed in the length of extraradical mycelium. Temperatures less than 18°C cause reduced growth and loosely connected and weak networks of mycelium. It also causes a delay in the formation of propagative and absorptive organs (Gavito et al., 2005). Carbon (C) is one of the most critical requirements for growth of AM fungi and it is strongly affected by soil temperature. Gavito et al. (2005) measured root-C uptake and observed C translocation in AM fungi is directly affected by variation in temperature. Lower temperatures (<18°C) slow down C translocation and affects the growth of fungi. Jennings (1995) documented the fluidity of membranes and movement of solutes were also negatively affected by low temperature.

Soil temperature, along with other abiotic factors such as soil pH, humidity, and amount of phosphorus (P), directly affects AM fungal spore density and colonization of roots (Alkobaisy, 2022). The occurrence and distribution of AM fungi is also widely affected by seasonal variation (Sivakumar, 2013). Seasonal variation not only restricts distribution of AM fungi but also affects infection ratio with associated plants (Bever, 2002). Fungal spore density is reported to be higher in dry seasons as compared to wet seasons (Hayman, 1970; Carvalho et al., 2001; Rajpurohit and Jaiswal, 2023). Glomalin, a mycorrhizal glycoprotein known for soil aggregation and structuring, are released more during dry seasons as compared to wet seasons (Vieira Junior et al., 2020). A decrease in glomalin content directly affects the activity of AM fungi and leads to less association with plants. Pirozynski (1981) reported that evolution of mycorrhizal association started in the tropics. Wang and Qiu (2006) discovered a wide distribution range of AM association in both tropical and temperate regions that is completely regulated by the presence of various abiotic factors.

Soil is an active biological device that regulates various essential biochemical reactions and ecological processes to support life. Microbes are an inseparable part of soil and widely contribute to its fertility. Soil properties like moisture, temperature, nutrients, and land-use system strongly influence the abundance and distribution of AM fungi (Ndoye et al., 2012). Siqueira et al. (1998) have reported that soil with low fertility increases the dependence of plants on mycorrhizal association. It is also observed that, under low soil fertility, fungal hyphae grow extensively inside the root (Sanders et al., 1977). Whereas degraded land, intensive agriculture practices, and urbanization lowers AM diversity and distribution (Brundrett and Abbott, 2002; Cardozo-Junior et al., 2012; Gupta et al., 2018; Flores-Rentería et al., 2020). Disturbance causes changes in plant communities and its species composition, which directly affects its infective potential and decreases spore density and root colonization (Birhane et al., 2020).

The AM association is found in all ecological niches of the world, but variation in altitude leads to considerable changes in AM fungal diversity and association. It may increase or decrease with changes in temperature, precipitation, nutrient availability, and plant community dynamics with altitude (Cotton, 2018). Yang et al. (2017) reported that altitudinal changes have a direct effect on diversity and relative abundance of soil fungi, which decreases a with decline in soil temperature. The diversity and dominance of AM fungi decreased with an increase in altitude (Gaur and Kaushik, 2012; Ghosh et al., 2012). AM fungal development is also influenced by the availability of hosts. Yang et al. (2016) reported that AM fungi show higher preferences toward plants of lower altitude than higher altitude. Less vegetational diversity and extreme environmental conditions of higher altitudes generally do not support AM association. Johnson (2010) observed that AM fungi diversity and association can be manipulated by altering resources that directly affect carbon demand and association.

A study of AM colonization in pteridophytes by Ghanta et al. (2016) reported that lithophytic pteridophytes have a higher percentage of root colonization, arbuscules, vesicles, and fungal spore count as compared to epiphytes. Arbuscules are found to be absent in the case of aquatic pteridophytes. Rare AMF colonization in epiphytic and aquatic species may be because AM fungi cannot be easily dispersed. Bajwa et al. (2001) studied AM association in wetland plants and observed this association begins in spring and reaches a maximum in summer, autumn, or winter, depending on the host species. Plant root morphology is yet another important factor that helps in the colonization of AM fungi (Baylis, 1975; Manjunath and Habte, 1991). Lycophytes and epiphytic ferns are known to have two types of root morphologies. Fat and fleshy roots are found to be colonized with fungi, whereas thin roots have smaller diameters, are thick-walled, have greater mechanical strength, and have a higher presence of phenolic compounds that are less susceptible to fungal association (Schneider, 2000; Pressel et al., 2016).

## Conclusion

Land plants are known to have diverged during the Neoproterozoic era from aquatic algae. This transition is considered to be one of the most significant turning points in the history of the Earth and evolution of land plants.

Pteridophytes are among the first vascular plants that invaded the terrestrial system after bryophytes. These non-seeded plants acted as pioneers, floor species, and primary producers in many of the ecosystems and facilitated the evolution of various other flora and fauna. They became a source of food and shelter for a variety of animals and acted as a favorable substratum for seedlings of higher plants. They played a very significant role in soil formation, restoration of habitat, and nutrient cycling. Symbiotic association with other microorganisms like cyanobacteria (nitrogen fixation) and mycorrhizal fungi (nutrient translocation such as Phosphorus) are some of the unique events that played a very important role in the early evolution and ecosystem functioning of the terrestrial system. Mycorrhizal association helped early land plants when conditions were not very favorable for survival. This association helped in nutrient translocation when availability was very limited and absorbing organs of plants were not very developed. AM association not only improved nutrient uptake but also made plants tolerant and resistant to various biotic and abiotic stresses. It has improved nutrient availability, structure, and fertility of soil, which later helped the rapid colonization of higher plants.

Climate change is a current and serious concern affecting natural ecosystems through and causing abiotic stresses like high temperature, salinity, flooding, and poor nutrient supply, in turn affecting plant growth and development. Pteridophytes have the potential to withstand such critical situations as many fossil records documented that, when life suffered major crises like mass extinction, pteridophytes colonized rapidly on barren lands and helped in the succession and establishment of new ecosystems. Mycorrhizal association played a very significant role in this process and helped pteridophytes to survive during unfavorable conditions by combating various stresses and helping to provide nutrition. In today’s time, when climate change is affecting ecosystem services, AM status needs to be better acknowledged in this group of plants. This review is a small attempt to gather information about AM-associated pteridophytes and fungal species, along with different AM structures like hyphae, arbuscules, and vesicles formed by them, which can be utilized in further research work.

## Author contributions

PK: Conceptualization, Resources, Supervision, Writing – original draft, Writing – review & editing. MB: Writing – original draft, Writing – review & editing. PG: Conceptualization, Writing – review & editing. PU: Conceptualization, Writing – review & editing.
